# Exploration of the viewing intention mechanism of Chinese traditional culture animated films based on SOR and TPB

**DOI:** 10.3389/fpsyg.2026.1797026

**Published:** 2026-04-16

**Authors:** Tianyang Yuan, Lin Lin, Xiao Xiao, Donglin Yang, Feng Su, Yan Li

**Affiliations:** 1 College of Arts, Northeastern University, Shenyang, China; 2 School of Design, Royal College of Art, London, United Kingdom; 3 School of Design Major in Industrial Design, Hanyang University, Seongdong-gu, Republic of Korea

**Keywords:** Chinese animation films, stimulus-organism-response (SOR), theory of planned behavior (TPB), traditional cultural symbols, viewing intention

## Abstract

The Chinese animated film market is developing fast and some of them have great success, such as “Nezha: The Monster Boy in the Sea” and the “Boonie Bears” series. But to understand the impact of traditional cultural symbols on viewing intent is underexplored. To bridge the gap between external cultural stimuli and internal cognitive evaluations, this paper conceptually integrates the Stimulus-Organism-Response (SOR) framework with the Theory of Planned Behavior (TPB). By positioning TPB’s psychological mechanisms (attitudes, norms, perceived control) as the ‘Organism’ state, this study constructs a theoretical model examining how traditional symbols drive viewing intentions, with cultural identity acting as a moderator. Surveying 441 Chinese animated film viewers, structural equation modeling indicates that traditional symbols positively impact attitudes, subjective norms, and perceived behavioral control, which subsequently drive the intention to watch films. Notably, attitude exerts the strongest effect on viewing intention, followed by subjective norms and perceived behavioral control. Cultural identity was found to moderate the relationship between attitude and intention, but it did not influence subjective norms or perceived behavioral control, indicating an asymmetric effect. Specifically, cultural identity enhanced the connection between attitude and intention without affecting the pathways related to subjective norms or perceived behavioral control. Additionally, a multigroup analysis (MGA) based on gender was conducted to confirm the model’s stability across different groups. Multi-group analysis verifies the structural invariance of the model across different genders, highlighting its robustness and general applicability. This study brings a new framework of understanding the consumption of cultural virtual products.

## 1 Introduction

Animation is a pillar industry in the cultural and creative sector, with the capability to boost new economic growth, drive cultural soft power for a nation and represents an important area of the global economy called “smokeless heavy industry”. It features cultural innovation, advanced technology and human intellectual labor. As people strive to raise their living standards and advance the economy, their cultural needs continue to evolve. The animation industry, as a vital sector of cultural creativity, is playing a more and more important role in economic growth (Fan and Feng, 2021). In recent years, traditional culture-themed Chinese animation films have experienced a surge in popularity at the box office. A seminal work in this respect is Monkey King: Hero Is Back (2015), generally considered a “milestone in domestic animated film production” (Jiang and Huang, 2017). Its smash hit represented a turning point for the domestic animation industry, which had long lain dormant, and rekindled expectations in audiences for homegrown works. The trend has further solidified with subsequent films, such as The Legend of White Snake (2019), Ne Zha 2 (2025), and Nobody (2025), which have delivered impressive box of-fice grosses and received critical acclaim. The success has not only revitalized the local animated film industry but also sparked renewed public interest in native animation, representing a significant and successful step in the evolution of China’s domestic animated film sector.

The existing literature provides extensive documentation on the factors that motivate audiences to watch mainstream movies. However, little is known about the factors that stimulate or discourage audiences from attending animation-based films. Most research efforts to recognize factors related to consumers’ intentions of viewing discard the term “films” as an all-inclusive class, instead of concentrating on one particular genre (like the animated film) (Shen and Hao, 2024; Haularizki and Rahayu, 2022). The Planned Behavior Theory (TPB) has become the primary theoretical model for much research on viewing intentions (Ramírez-Castillo et al., 2021; Zhang and Yoon, 2018; Mataracı and Kurtuluş, 2020). These analyses usefully demonstrate the relevance and logical consistency of using TPB within the context of viewing intentions. They hypothesize a positive relationship be-tween attitudes, subjective norms, and perceived behavioral control and consumer intention to view films. Unfortunately, the constraint lies in the fact that the generality of those three variables is still non-specialized. Since other factors are not being considered (psychological variables only), it is limited to predicting the film-viewing behavior. A new theory must then be developed from the perspective of integrating other elements, such as traditional Chinese cultural symbols, so that all the external causes may render a complete explanation thereof. Stimulus–organism–response (SOR) theory posits that external environmental cues affect internal psychological states, which subsequently drive behavioral responses. While TPB effectively models rational decision-making through attitudes, norms, and control, it lacks a mechanism to explain how external cultural elements trigger these internal evaluations. By embedding TPB within the ‘Organism’ component of the SOR framework, this study bridges this theoretical gap. We propose that traditional cultural symbols act as salient external stimuli (S) that activate the specific psychological evaluation processes (O) delineated by TPB, ultimately driving viewing intentions (R) in a culturally embedded context.

According to structural equation modeling of 441 valid samples, three contributions have been made in this study: Firstly, “traditional cultural symbols” were firstly defined as external stimuli (S) of cultural virtual products in SOR, which expands the application scope of the theory in cultural virtual product consumption. Second, build and test an integrated SOR-TPB serial mediation model, find out the psychological process for turning external cultural signs into viewing intention and fill in the research gap. Third, it also finds that cultural identity only moderates the attitude-viewing intention path but does not moderate the subjective norms or perceived behavioral control paths. Rather than challenging the implicit assumptions of previous TPB studies, this asymmetric boundary condition refines the model’s contextual application by demonstrating that cultural factors primarily amplify affective evaluations rather than social or practical constraints.

## 2 Literature

### 2.1 Stimulus-organism-response theory (SOR)

Based on behavioral psychology, the stimulus-organism-response (SOR) theory by Mehrabian and Russell (1974) suggests that SOR is a progressive sequence in such a way: ‘Stimulus (S) → Organism (O) → Response (R)’. According to this theory, external input triggers a set of an individual’s cognitive and affective processes (the organism), which then bring about either approach or avoidance actions (the response) (Jacoby, 2002). The SOR model has been widely used in various domains, including social commerce (Wu and Li, 2018), online shopping (Aggarwal and Rahul, 2017), website design (Molinillo et al., 2021), and text-image-based online reviews (Bigne et al., 2020). It has received approval from a wide range of researchers as the basis for analysing and understanding user behaviors. The SOR model has also been extensively confirmed in relation to media consumption and the analysis of cultural products. In this respect, the stimulus refers primarily to social media advertisements featuring cultural products (Hew et al., 2018), product images (Kim et al., 2020), and their cultural expressions (Liu and Zhao, 2024). As for the organism, it mainly reflects consumers’ cultural identity with the product (Kim and Heo, 2021), flow experience (Ouyang et al., 2024) and psychological identification (Yang et al., 2022). The answer primarily reflects consumers’ behavior toward the cultural product, such as their satisfaction degree (Kim and Heo, 2021), attention to film products (Zhang and Yoon, 2018), and purchase intention (Guo et al., 2021).

Most studies using the SOR model have focused on the purchase intentions of physical products, while ignoring virtual cultural products, such as movies. Fu et al. (2018) extended the SOR model and situation cognition theory to explain the intentions of viewing by combining with trust transfer and information technology acceptance theory. Their results highlight the significant effects of external and internal similarity on perceived usefulness, perceived enjoyment, and trust transfer for users’ decisions to purchase movie tickets. Additionally, May et al. argued that the quality of the content and its accessibility would ultimately determine OTT viewers’ perceived enjoyment of OTT and their attitudes, which in turn would impact consumers’ satisfaction and loyalty towards these platforms. However, little is known about the intention to watch Chinese animation films. To fill the gap, this paper adopts the SOR model to explore viewing intention toward Chinese animation films, which are representative of external stimuli with traditional cultural symbols. Symbolic cues can eventually evoke psychological processes termed the “organism (O),” including cultural identity, attitudes, subjective norms and perceived behavioral control. In the context of this study, “response (R)” refers to consumers’ intent to visit cinemas for Chinese animation film screenings. The SOR framework explains the processes of developing viewing intentions towards Chinese cultural symbols by connecting external stimuli to personal emotions and cognition. This relationship deepens the understanding of reward incentives and mechanisms in an environment of increased cultural confidence, providing theoretical references for relevant research and practices.

### 2.2 Traditional cultural symbols in Chinese animation and viewing intention

Traditional cultural symbols include linguistic and non-linguistic ones, and are “bearers of culture”. They are central to cultural production, the circulation of cultural meaning and inheritance (Rosendaal and Reitsma, 2017). The study of culture-specific symbols is a branch of modern semiotics (Zong et al., 2023) that encompasses research in diverse areas, including folklore studies, anthropology, narratology, revelatory analysis, and artistic semiotics (Greimas, 2002). In this regard, cultural symbols represent core systems of meaning by which cultural communities define themselves. At the same time, they are representative systems that other cultural groups can learn to understand and engage with (Read, 2014). Traditional Chinese cultural symbols are indelible elements; they are the carriers of national psychology. They are outward expressions of the varied ideological cultures and philosophies that have shaped the nation’s history (Jianchao, 2023). The Wan Laiming brothers in China have led the Chinese animation industry, and the nation has pursued nationalization of the industry. Pertains to works like Journey to the West and Nezha Conquers the Dragon King, which contain significant cultural elements in animation. Concrete symbols encompass ink-wash painting styles, ancient architectural designs, traditional craft patterns, and so forth; abstract symbols range from Confucian and Taoist thought, folk customs, to mythological narrative prototypes. Although these symbols span diverse dimensions—from visual aesthetics to deep semantic narratives—they collectively operate as a cohesive cultural stimulus in the context of animation. Consequently, this study operationalizes traditional cultural symbols as a multidimensional yet unified latent construct, representing the holistic “Chinese aesthetic and cultural” experience perceived by the audience. Such symbols are incorporated into animation through traditional Chinese folk arts, including painting, paper-cutting, and theatre. As a result, Chinese animations have long become an important channel for conveying and spreading traditional culture symbols. With the ‘national trend’ phenomenon and cultural heritage protection policies, traditional cultural elements have been integrated into Chinese animation films, resulting in a surge of more creative works in recent years. Instead of existing solely as superficial remixes of parts, they are now visually integrated into narratives, personas and themes. The integration not only promotes the sense of cultural identity among the visiting community and facilitates interaction between different cultures, but also stimulates cultural creativity by providing new inspiration for inheriting intangible cultural heritage.

Like any other cultural industry, cinema is composed of material goods that are planned, produced, and marketed to convey symbolic content. Its programming is influenced by the institutional structures of the economic, cultural, and political organizations in each country of the region, which collaborate with sectors such as culture, social work, and entertainment (Peattie and Peattie, 2009). In particular, the significance of marketing is quite high when it comes to a film’s opening weekend, as consumers’ perceived value and benefits of watching movies in theatres play a significant role in their purchase intention (Mehrabian and Russell, 1974). Today, academic studies on intentions to watch are still predominantly focused on the movie as a whole. Production cost, story, and the director’s name are key determinants in predicting the viewing interest of potential consumers (Sardanelli et al., 2019). Craig et al. (2015), Liu (2006), and Breves and Schramm (2019) revealed that variables such as spotting sound effects, visual effects, plot importance, and film enjoyment influence consumers’ evaluations of films and their word-of-mouth.

In contrast, previous studies of Chinese animation films have mainly focused on artistic expression. In their semiotic interpretation of the 21st-century Chinese child-hood image and the conflicts between individual agency and social convention, as exemplified in the animated feature Ne Zha, Chen and Lau (2021) discussed the impact of intersemiosphere from Chinese culture. Jiang (2024) has evaluated the importance of China’s animation industry and the effective factors in explaining target market preferences, focusing on visual attraction, character representation, and story lucidity, by employing the FAHP and GRA methods. The current study demonstrates the usefulness of hybrid MCDM approaches for Chinese animation companies to ad-just their internal resources more closely towards audience expectations and, ultimately, to make informed decisions. A few similar studies, such as Wu (2024), have adopted the Theory of Planned Behavior to investigate Chinese youth’s intention to watch traditional cultural literature-based animated films. This study identified the following factors as significant: educational level, income level, perceived behavioral control, attitudes, and subjective norms, with no significant effects observed from gender. However, researches with such designs focus solely on isolated “traditional symbols” in animations, ignoring the application of psychological or behavioral theories that can provide a good explanation for the interaction mechanism between the symbol and the audience. This lack of reflection obstructs the direction of the generation and development of Chinese animated films.

### 2.3 Planned behavior theory (TPB)

The theory of planned behavior (TPB) was proposed by Ajzen (1991) and is a widely used theoretical basis for predicting attitudes. It posits that behavioral intention is a function of three fundamental constructs: attitude, subjective norm, and perceived behavioral control. Attitude is what an individual thinks about performing a behavior favourably or unfavourably. Subjective norm refers to the perceived social pressure from others (particularly close others, such as family and friends) to engage in or refrain from engaging in a particular behavior. Perceived behavioral control reflects one’s perception of the ease or difficulty of performing the behavior, as well as the perceived availability of resources, time, and abilities. The theory posits that the more one intends to perform a particular behavior, the stronger the likelihood that it will be executed. The TPB provides a useful model to understand the psychology behind user and their behaviors. It has been used to analyse the behavior of people in various contexts, including predicting users’ purchase intentions. Most existing investigations focusing on the effects of personal norms upon movie-watching intentions also utilize TPB (Mehrabian and Russell, 1974). Among consumer markets, perceived behavioral control, social network, and consumer attitudes are the variables used to explain a directly influenced dependent variable of the green purchasing intention. Cinema, according to the Theory of Planned Behavior, provides consumers with a pleasurable experience that influences their attitudes towards film viewing and hence theatres, wherein they esteem cinema-going as a desired activity (Mataracı and Kurtuluş, 2020). Researchers Ramírez-Castillo et al. (2021), and Zhang (2025) employed the Theory of Planned Behavior (TPB) to investigate various dimensions of film-viewing intentions. Although the theory is widely used, some scholars have raised substantial doubts about its capacity to account for the heterogeneity of human intentions and behaviors (Ajzen, 2002; Rhodes and Courneya, 2003).

Furthermore, the classical TPB model exhibits some deficiencies in predicting the intention to watch Chinese animation. First, it highlights only the general psychological factors of attitudes rather than constructs associated with the unique cultural context of Chinese animation. Second, previous applications of TPB in animation research specifically concentrated on “general video viewing,” unable to establish constructs that can be used to reflect the cultural specificity of Chinese animation. Consequently, the contextualized adaptation was not sufficient. The main components of the TPB (attitude, subjective norm, perceived behavioral control) are included in this study within the Stimulus-Organism-Response architecture, representing the ‘organism’ (O). This integration enables a better fit with the psychological mechanisms underlying the viewing intentions for Chinese animation. That is, attitude is viewers’ evaluation of the importance of watching Chinese animation; subjective norm is the social pressure that peers and family members put on the individual with respect to animation consumption; perceived behavioral control considers individuals’ perception about whether they have time to spare for watching Chinese animation and whether they have ways to do so. Together, these three elements contribute to the concept of viewing motives.

## 3 Hypothesis development

### 3.1 The impact of traditional cultural symbols on the organism (O)

Within the SOR framework, the ‘Organism’ represents the internal psychological states triggered by external stimuli. TPB constructs (attitude, subjective norms, and perceived behavioral control) appropriately encapsulate this state, as they translate external cultural stimuli into structured cognitive and normative evaluations before behavioral execution. Chinese traditional culture, including calligraphy, paper-cutting, traditional pat-terns, and festival atmosphere, is an expression of national emotions. Chinese traditional culture, including calligraphy, paper-cutting, traditional pat-terns, and festival atmosphere, is an expression of national emotions. When such symbols are visually highlighted or contextually backgrounded, they may evoke consumers’ cultural identity and emotional bonding, thereby promoting favourable attitudes. In terms of visual appearance, cultural symbols have their own advantages in enhancing the expressive and ornamental capacity. From a design perspective, incorporating traditional cultural items can provide intuitive and easily perceivable communicative advantages, greatly reducing the likelihood of misunderstanding cultural implications. In a market with homogenized products, an item that represents real cultural aspects is more likely to be noticed by the consumer as a differentiated product (Li et al., 2022). Current research indicates that the more individuals are culturally embedded in their culture, the more favourable their attitudes are toward its derived products and behaviors, resulting in positive consumer evaluations (Zheng et al., 2022). Therefore, we hypothesize:

*H1*: The presentation of traditional Chinese cultural symbols will significantly and positively influence consumer attitudes.

Subjective norms refer to the perceived social pressures or intentions of others regarding particular behaviors. Chinese traditional cultural symbols are not only the embodiment of national culture but also closely tied to the social credit system and collective identity. When people come across these symbols in social or consumption circumstances, they might feel positive normative prospection from the group, family or society, and this may lead them to either comply with (or support) the linked attitudes. For instance, Shimp and Sharma (1987) noted that consumers exhibit a preference for domestic brands over foreign brands when they are faced with external competition from outside their group. The subtle or overt inquiries from important others and cultural communities strengthen people’s beliefs in subjective norms. Therefore, we hypothesize:

*H2*: The presentation of traditional Chinese cultural symbols will significantly and positively influence consumers' subjective norms.

Perceived behavioral control is defined as an individual’s belief about their ability to perform a given behavior—cultural confidence and self-efficacy in heritage cultural values, accompanied by corresponding cultural pride. Traditional Chinese culture symbols are often associated with expressions of collective value reaffirmation, reinforcing the source group’s control and mastery over these relevant identities. In the field of product design, incorporating elements of traditional culture can ensure that objects possess layers of cultural significance, transforming ordinary commodities into rich cultural experiences that influence value perception (Slobin, 1992). If the consumer encounters symbols from their environment in products or environments (and if they seem familiar and meaningful), they are more willing to believe that they can understand, use, or interact with it—they perceive behavioral control (Zhang et al., 2020). Consequently, we hypothesise:

*H3*: The presentation of traditional Chinese cultural symbols will significantly and positively influence consumers' perceived behavioral control.

Attitude is a central variable in the TPB. As a psychological process, it permits individuals to develop persistent attraction or repulsion to certain agents (Eagly and Chaiken, 2007). In relation to this study, attitude represents a respondent’s positive predisposition for “going to cinemas to watch Chinese animation films that involve traditional cultural symbolic elements”. Previous studies have shown that attitude has a significant positive effect on behavioral intention toward purchasing organic foods (Yazdanpanah and Forouzani, 2015; Konuk, 2018; Lee and Yun, 2015) and green products (Alam et al., 2024; Demir et al., 2021; Zhang et al., 2019). Similarly, various studies have highlighted the importance of consumer attitudes in influencing their intention to view movies. Indeed, researchers have used TPB to embed the model within their own research contexts and have found that consumer attitude directly correlates positively with purchasing intention for green products (Mehrabian and Russell, 1974; Ajzen, 2002; Li et al., 2022; Vergara et al., 2019; Simamora and Djamaludin, 2020). From this existing literature, the following hypothesis is developed:

*H4*: Attitude has a significant positive influence on an individual's intention to watch Chinese animation films that incorporate traditional cultural symbols.

### 3.2 TPB constructs (O) and the intention to watch Chinese animation films

The social pressure felt by a person when deciding whether or not to perform a behavior (Ajzen, 1991). This integration process has been empirically demonstrated to have a significant impact on an individual’s behavioral intentions, attitudes, and corresponding behaviors. It is based on the perception of “significant others” (family, friends, classmates) that an individual should act in a certain way. After that, the person will finally accept it for themselves (Al-Swidi et al., 2014; Tan et al., 2022). Within academic discourse, this social norm is also described as people’s normative beliefs, i.e., the subjective perception of the extent to which important reference persons (for instance, friends, family members, partners or colleagues) approve or disapprove of specific behaviors (Si et al., 2020; Kumar et al., 2021). One of the most important psychological aspects of close social relations is Word-of-Mouth communication, which also involves subjective norms (Sweeney et al., 2014). They all play an equally significant role in individual behavior.

Within the context of film consumption, an individual’s belief that other important people will have a favorable opinion about watching a movie is significantly associated with intention to view (Villar et al., 2019; Vergara et al., 2019). This opinion is confirmed by available evidence: if one’s friends, family, or workmates recommend a film, the intention to watch it rises, and the intensity of that intention increases with the closeness of the friend, family or workmates. In addition, group members who have a professional connection but no strong personal relationship based on film criticism (Fogel and Prabhu, 2022; Pang et al., 2022; Feng et al., 2020) can still increase movie consumers’ viewership intentions to some extent when their opinions offer social support via online information platforms (e.g., film review websites), thereby alleviating temporal and spatial constraints in acquiring information (Zhang and Chen, 2022; Fogel and Prabhu, 2022).

Based on such theoretical and empirical bases, with attention to the particular research subject matter of “Chinese animation films embodying traditional cultural symbols”, this hypothesis is put forward as:

*H5:* Subjective norms exert a significant positive influence on viewing intentions for Chinese animation films containing traditional cultural symbols.

Perceived behavioral control is one of the three variables within TPB. It is the degree to which an individual feels in control of their ability to enact a certain behavior. People are more likely to be motivated to engage in a behavior when they perceive the necessary resources or opportunities to perform it without interference (Zhang, 2024; Paul et al., 2016; Dorce et al., 2021). Preliminary studies have revealed three major PBC subdimensions: physical inconvenience, time costs, and resource availability (Wang et al., 2014). In creating movies, perceived behavioral control has a significant effect on the intention to view (Mataracı and Kurtuluş, 2020). It is empirically proven that an audience with more leisure time and disposable income is more willing to watch movies (Mehrabian and Russell, 1974).

The finding is consistent with studies developed in the context of China, where perception of behavioral control is a predictor of intention to watch animation movies in cinemas (Li et al., 2022) among Sichuan University students’ intention to view traditional literature-adapted animation films (Ajzen, 2002). Furthermore, the results reveal that consumers’ perceived behavioral control towards released movies is related to film quality, as well as word-of-mouth feedback about films from fans and critics, and box office returns (Chiu et al., 2019). Motivated by an analysis of the relevant academic literature, we hypothesize:

*H6*: Perceived behavioral control exerts a significant positive influence on audiences' intention to view Chinese animation films incorporating traditional cultural symbols.

### 3.3 Moderating role of cultural identity

A few studies suggest that consumers’ cultural identification may increase their feelings of cultural belonging and perceived value towards the product, which, in turn, may directly or indirectly affect their purchase intention. Cultural identity is defined as the psychological relationship between one’s selfconcept and a particular culture. Another view emphasizes the evaluative aspect of cultural identity, conceptualizing it as the extent to which an individual endorses particular cultural features, such as making positive associations between traits and values associated with a certain group. Furthermore, Wang and Hu (2011) classify cultural identity into the following three aspects: cultural symbol identity, cultural identity, and cultural value identity. Cultural symbol identification is a process of evaluating an individual’s attitude orientation toward symbolic expressions, such as material forms, language, and life events, in various cultural settings. Cultural identity measures the degree to which individuals practice their attitude, feel at home, and share agendas with culturally diverse groups. Lastly, cultural value identification is interpreted as an indicator of one’s own knowledge or awareness of the acceptance of social norms and values typical for a given culture.

Some scholars within consumer behavior research suggest that the patient components of TPB—i.e., individual attitudes, subjective norms, and perceived behavioral control—are insufficient to explain consumer purchasing decisions. For instance, Simonson (1992) extended the original TPB model by incorporating variables such as cultural identity and moral norms, thereby demonstrating that cultural identity has a significant impact on the ‘attitude-behavior’ link. Likewise, Shimp and Sharma (1987) found that consumers often prefer domestic brands over foreign alternatives, especially when faced with a choice between local and global options. This preference is even more pronounced for the consumer whose in group faces threats from outsiders.

As discussed above, traditional culture symbols lead to greater emotional intensity and cultural identification for consumers. “As institutions that transmit knowledge, beliefs, values, attitudes, traditions and lifestyle (members) being part of a group, the basis is established for building cultural identification (Chang et al., 2021).” This research suggests that the mediation of traditional cultural symbols in Chinese animation on individuals’ identification with their own culture is influenced by their positive evaluation of the core expressive and deep-layered meaning connoted in such symbol designs, which are shaped by the national ideological situation overall. Moreover, this cultural identification creates interest, pleasure and a sense of belonging (Liu et al., 2023) that strengthens organismic cognition toward a culture embedded in the animated products. Hereby, organismic cognition refers to the primary cognitive processing of traditional cultural symbols as being displayed in animation.

Additionally, the influence of organismic cognition on viewing intention may vary depending on the strength of cultural identity. When consumers have a high level of cultural self-identity, they are more likely to perceive the value of traditional cultural symbols in organismic cognition, thereby strengthening their beneficial effect on viewing intention. In contrast, such an influence is relatively lower when cultural identity is low. So, this study suggests the following hypotheses:

*H4a*: Cultural identity has a positive moderating effect on the relationship be-tween consumers' attitudes and their viewing intentions.

*H5a*: Cultural identity positively moderates the relationship between consumers' subjective behavioral norms and their viewing intentions.

*H6a*: Cultural identity positively moderates the relationship between consumers' perceived behavioral control and their willingness to watch films.

**Figure 1:**
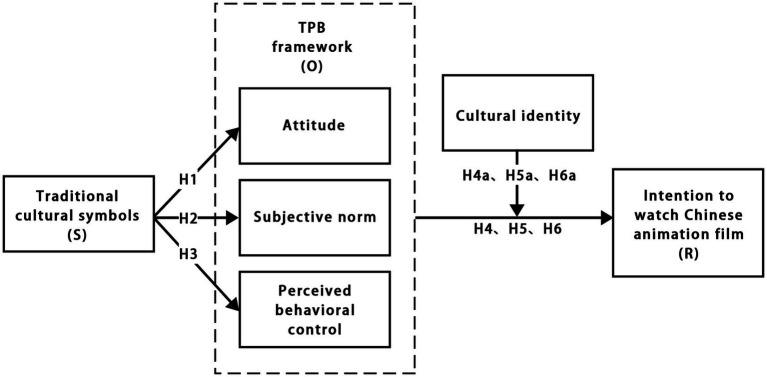
The conceptual framework of the research.

In summary, this study synthesizes pertinent literature to develop a theoretical model. The model’s core variables encompass traditional cultural symbols, organismic factors—such as attitude, subjective norms, and perceived behavioral control—cultural identity, and the willingness to view films. Figure 1 provides a detailed representation of the theoretical model.

## 4 Methods

### 4.1 Variable measurement and questionnaire design

To ensure the reliability and validity of the study, this paper presents the translation of foreign scales into Chinese as research tools for the measurement items, which are adapted from well-known domestic and international scales. Focused changes were then made based on three main aspects: firstly, consistency with the characteristics of traditional cultural symbols; secondly, enhancement of question readability and clarity of item wording; and thirdly, use of the question as a declarative to ensure accurate questionnaire item design. Respondents’ opinions on each statement were assessed using a 5-point Likert scale. For the assignments, a numeric value will represent each answer: 1 for Strongly Disagree, 2 for Disagree, 3 for Undecided, 4 for Agree and Clear, and finally, 5 for Strongly Agree. The answer options 1–5 represented the degree of agreement in each response according to the respondent’s own belief and experience.

This paper analyzes the path by which traditional Chinese culture symbols influence consumers’ willingness to watch movies. For measurement items, traditional cultural symbols in Chinese animation films were primarily identified through a review and synthesis of available literature. Traditional cultural symbols (TCS) awareness was measured using a seven-item scale, and cultural Identification (CI) is determined by five items from Zong et al. (2023) scale. The scale items for all three constructs, i.e., ATT, SN and PBC, are taken from various pre-tested scales present in the literature (Qi and Ploeger, 2021; Imani et al., 2021; Ulker-Demirel et al., 2018). Consumer intention to view (INT) was assessed with four items taken from Ramírez-Castillo et al. (2021). To profile the demographic background of respondents, additional items were appended to the questionnaire based on the design approach of Zhang and Zhang (2025). All variables collected in this research were suitably captured by the items already presented. The scale content of each variable is presented in Table 1.

**Table 1:** Model measurement list.

Dimension	No.	Scale items	Source
Traditional cultural symbols	TCS1	I can recognize common traditional cultural symbols (e.g., patterns, rituals) in Chinese anfZongimation films.	Zong et al. (2023)
	TCS2	The presentation of traditional cultural symbols in Chinese animation films is visually attractive to me.	
	TCS3	I understand the historical background behind the traditional cultural symbols in Chinese animation films.	
	TCS4	I can grasp the philosophical ideas conveyed by traditional cultural symbols in Chinese animation films.	
	TCS5	I am familiar with the cultural meanings of language-related symbols (e.g., proverbs, calligraphy) in Chinese animation films.	
	TCS6	I prefer Chinese animation films that contain rich traditional cultural symbols.	
	TCS7	I know well about folklore-related symbols (e.g., myths, festivals) in Chinese animation films, and I actively learn their connotations.	
Cultural identity	CI1	Watching Chinese animation films with traditional cultural symbols makes me feel a strong connection to my cultural identity.	Zong et al. (2023)
	CI2	I believe that traditional cultural symbols in Chinese animation films are an important part of our cultural heritage.	
	CI3	I often share my insights about traditional cultural symbols in Chinese animation films with my peers.	
	CI4	I like comparing traditional cultural symbols in Chinese animation films with those from other cultures to find differences.	
	CI5	I will prioritize watching Chinese animation films with traditional cultural symbols when I go to the cinema.	
Attitude	ATT1	Watching Chinese animation films with traditional cultural symbols in the cinema makes me feel excited and engaged.	Qi and Ploeger (2021) and Imani et al. (2021)
	ATT2	I believe that Chinese animation films with traditional cultural symbols are valuable and meaningful works worth watching in the cinema.	
	ATT3	Compared to other types of films, I prefer to watch Chinese animation films with traditional cultural symbols in the cinema.	
	ATT4	I would actively choose to watch Chinese animation films with traditional cultural symbols when I go to the cinema.	
Subjective norm	SN1	My family’s preference for Chinese animation films with traditional cultural symbols influences my choice to watch them in the cinema.	Ulker-Demirel et al. (2018)
	SN2	Positive reviews of Chinese animation films with traditional cultural symbols on social media make me more willing to watch them.	
	SN3	Recommendations from influencers or celebrities make me more likely to watch Chinese animation films with traditional cultural symbols.	
	SN4	Most people around me think that watching Chinese animation films with traditional cultural symbols is a meaningful choice.	
Perceived behavior control	PBC1	I have full control over whether to watch a Chinese animated film with traditional cultural symbols.	Ulker-Demirel et al. (2018) and Qi and Ploeger (2021)
	PBC2	If the Chinese animated film with traditional cultural symbols I want to watch is not playing in my nearby cinema, I would go another cinema showing it.	
	PBC3	I am willing to pay a reasonable price to watch a Chinese animated film with traditional cultural symbols in the cinema.	
	PBC4	Even if my schedule is tight, I can arrange time to watch a Chinese animated film with traditional cultural symbols in the cinema.	
Movie watching intention	INT1	If I need to choose between a Chinese animated film with traditional cultural symbols and a foreign animated film today, I will choose the former.	Mehrabian and Russell (1974)
	INT2	If I were to go to the cinema this week, I would prioritize watching a Chinese animated film with traditional cultural symbols.	
	INT3	I plan to watch multiple Chinese animated films with traditional cultural symbols in the cinema in the future.	
	INT4	I am more willing to recommend Chinese animated films with traditional cultural symbols to others than other types of films.	

### 4.2 Data collection

The questionnaire was initially developed for this study. In accordance with the design of the model and research object, a questionnaire survey was conducted to investigate the samples. The subjects involved people aged 18 and above, who might have been exposed to Chinese traditional culture animation and other concerned persons. The survey was designed and distributed using an Internet-based questionnaire. The questionnaire consisted of two parts. Section one collected the respondents’ demographics (gender, age or educational background). Secondly, the study investigated participants’ feelings towards Chinese animation films along four different perspectives (traditional cultural symbols, organism states, cultural identity and viewing intention).

A pilot survey was conducted with a small group of respondents before the main survey. Findings from this pilot study were used to help strengthen and refine the research. The primary survey was conducted from September 7 to October 7, 2025. Additionally, a detailed explanation was provided to clarify the study’s aim, and written consent was obtained from all participants. We obtained 453 questionnaires with the SurveyStar platform, and among them, 441 were considered valid. The demographic characteristics of the respondents were analyzed descriptively and statistically using SPSS Statistics 26.0. This description used numerical data (frequency, percentage) to describe the participants and simplify the interpretation of subsequent inferential results. The results of this survey are shown in Table 2.

**Table 2:** Participants’ profile.

Measure	Questionnaire items	Frequency (*N* = 441)	Percentage (%)
Gender	Male	242	54.88
	Female	199	45.12
Age (years)	18–25	327	74.15
	26–35	64	14.51
	36–45	25	5.67
	Over 46	25	5.67
Education	Secondary education	203	46.03
	University education	127	28.80
	Master’s or PhD	111	25.17
Monthly income (RMB)	Under 2000	214	48.53
	2000–5,000	98	22.22
	5,000–8,000	80	18.14
	Over 8,000	49	11.11
Total		441	100.00

### 4.3 Data analysis

Because the research data are based on self-reporting of surveys in a single questionnaire, there is a risk of common method bias (CMB). So it is got procedures and statistics. First, many procedural steps were used to reduce the possibility of bias such as making the entire questionnaire completely anonymous, stating clearly that there is no right or wrong answer, separating items of the predictive and dependent variables in different parts of the questionnaire, randomizing the order of some items, using unambiguous language, and providing clear instructions at the beginning of the questionnaire to reduce the likelihood of social desirability bias. And these would also lessen general appraiser results, item context results, and fleeting emotional impacts.

Statistically, this study initially employed Harman’s single-factor test to assess common method bias. The results showed that all items loaded on a common factor, and the first factor extracted from the non-rotated solution explained only 38.2% of the total variance, which is well below the 50% threshold. However, it is widely acknowledged in academic literature that Harman’s test can be insensitive in detecting moderate levels of bias. To address this limitation and provide a more robust validation, we supplemented this analysis with two advanced techniques: the unmeasured latent method factor (ULMV) and the theoretically unrelated marker variable method.

In addition, unmeasured latent variable factor (ULMV) was used, and an orthogonal and unrelated method factor was added to the confirmatory factor analysis measurement model. The results indicated that the model fit indices only slightly changed pre- and post-introducing the methodological factors (ΔCFI = 0.006, ΔTLI = 0.005, ΔRMSEA = 0.002, ΔSRMR = 0.004). Methodology-related factors explained a small percentage (4.7%) of the common variance, which is well below the 25% of methodological factors suggested by Williams et al. The result also shows that the common method bias is not statistically meaningful; it did not have any important effect on the conclusion. And, in order to strengthen the robustness of this test, a theoretically unrelated marker variable (3 items: “liking blue color”) was also included. The analysis of the marker variables yielded low correlation coefficients (mean r = 0.052) with the main constructs. Compared with the unadjusted model, the adjusted path coefficients changed little (Δ*β* < 0.05), and the significant paths were still significant. Thus, it was proved that there was no common method bias threatening the research results. In short, after many tests, the data quality of this study is good enough and can go on to more analysis. In short, after many tests, the data quality of this study is good enough and can go on to more analysis. Furthermore, to test the moderating effect of cultural identity, a product-indicator approach was utilized to construct latent interaction terms in the subsequent structural equation modeling.

## 5 Results

### 5.1 Descriptive analysis

Regarding gender variables, males comprised 54.88% of the sample, while females accounted for 45.12%. This relatively equal distribution of males and females further increases the representativeness of this sample. In terms of age, 74.15% were between the ages of 18–25, 14.51% were between the ages of 26–35, 5.67% were between the ages of 36–45, and a small percentage were aged over 46 years. In terms of education, secondary school (46.03%) is the most common, followed by university studies, both at undergraduate (28.80%) and graduate level (Master’s or PhD: 25.17%). As for monthly income, most reported having no income, followed by <¥3,000 per month. This ratio indicates a higher proportion of high school and the new working generation in the sample. These characteristics align with young consumers, who are willing to try new experiences and are involved in individualized, experience-based, and pleasure-seeking consumption.

### 5.2 Assessing the measurement model

To guarantee the reliability and validity of the measuring instrument, the reliability assessment, validity assessment (both convergent validity and First, we adopt the Fornell-Larcker criterion: the square roots of the AVEs of each construct were above the correlation coefficients with all other constructs (lower triangles of Table 3), meaning that the constructs were relatively well differentiated from each other. Secondly, we use the Heterotrait–Monotrait (HTMT) ratio method), and overall testing of the measurement model were carried out. All the data are based on 441 valid samples. Using Amos 24.0, we conducted CFA validation. Reliability tests were performed. Internal consistency reliabilities of the six latent variables of this study: traditional cultural symbols (TCS), cultural identity (CI), attitude (ATT), subjective norms (SN), perceived behavioral control (PBC), and viewing intention (INT). It is evaluated by Cronbach’s alpha and composite reliability (CR). The results show that the Cronbach’s a coefficients for each dimension were 0.880–0.953, which is greater than the recommended value of 0.7 by Nunnally (1975). Therefore, it can be said that the scales possess adequate internal consistency. Meanwhile, the CR values for each dimension was from 0.878 to 0.953, which was also higher than the CR cut-off point at 0.70, thus further confirming the reliability.

**Table 3:** Discriminant validity (Fornell-Larcker criterion, correlations, and HTMT).

Construct	TCS	CI	ATT	SN	PBC	INT
Traditional Cultural Symbols (TCS)	0.862					
Cultural Identity (CI)	0.450	0.837				
Attitude (ATT)	0.420	0.480	0.824			
Subjective Norm (SN)	0.380	0.410	0.550	0.802		
Perceived Behavioral Control (PBC)	0.400	0.390	0.520	0.490	0.846	
Intention (INT)	0.440	0.460	0.580	0.510	0.600	0.848
HTMT values (above diagonal)		0.712	0.698	0.642	0.668	0.724
			0.756	0.678	0.645	0.742
				0.812	0.778	0.798
					0.732	0.756
						0.810

Assessment of convergent validity. Convergent validity was mainly tested by standardized factor loading and Average Variance Extracted (AVE). The standardized factor loadings for each item ranged from 0.770 to 0.872, and all loadings were significant (*p <* 0.001) and higher than the 0.70 threshold suggested by Hair et al. (1998), indicating that the items had strong explanatory power for the corresponding constructs. In addition, the AVE values of the constructs ranged from 0.643 to 0.743, all of which were greater than the 0.50 criterion suggested by Fornell and Larcker (1981), which meant the constructs could easily pull out the underlying traits measured by the items. Therefore, the First, we adopt the Fornell-Larcker criterion: the square roots of the AVEs of each construct were above the correlation coefficients with all other constructs (lower triangles of Table 3), meaning that the constructs were relatively well differentiated from each other. Secondly, we use the Heterotrait–Monotrait (HTMT) ratio method was good.

In order to make sure of the discriminant validity, the researcher used a lot of different ways. First, we adopt the Fornell-Larcker criterion: the square roots of the AVEs of each construct were above the correlation coefficients with all other constructs (lower triangles of Table 3), meaning that the constructs were relatively well differentiated from each other. Secondly, we use the Heterotrait–Monotrait (HTMT) ratio method: The HTMT values were all lower than the conservative threshold of 0.85 (up to 0.812), and none of the 95% confidence intervals of the HTMT inference test contained 1, which further confirmed that there were clear distinctions among the constructs. Finally, a cross-loading test was carried out, and it was found that all items had far higher loading on the construct it was related to compared to all other constructs, no cross-loading issues. This consistent with these multiple criteria to support the good First, we adopt the Fornell-Larcker criterion: the square roots of the AVEs of each construct were above the correlation coefficients with all other constructs (lower triangles of Table 3), meaning that the constructs were relatively well differentiated from each other. Secondly, we use the Heterotrait–Monotrait (HTMT) ratio method of the measurement model in this research. The fit of the measurement model was judged by means a number of often used indicators in the fit. Results show that the chi-squared to degrees of freedom ratio (CMIN/df) = 1.461, which is smaller than the tight criterion of 5; the root mean square of approximation error (RMSEA) = 0.032, which is lower than the suggested value of 0.080; the comparative fit index (CFI) = 0.979 and the Tucker-Lewis index (TLI) = 0.977, both of which are greater than the criterion of 0.900; The goodness-of-fit index is GFI = 0.908, and the normativeness fit index NFI = 0.936 and the adjusted goodness-of-fit index AGFI = 0.894, all of which met or exceeded the recommended thresholds of Hu and Bentler (1999) and Jöreskog and Sörbom (1993). Taken together, these metrics indicate that the measurement model fits the observed data highly and that the model structure is stable and reliable.

### 5.3 The structural model

In order to ensure the rigor of the establishment of the model, this paper will construct the structural model strictly according to the theoretical framework of SOR and TPB. External stimuli (traditional cultural symbols) directly impact the state of the organism (attitude, subjective norms, and perceptual behavioral control), then the state of the organism impacts the response (intention to watch a film). Cultural identity as a moderating variable is only put on the road from attitude to viewing intention (both based on theoretical derivation and empirical results, there is no significant moderating effect on the other paths).

The model does not have improper paths or loops, it is recognizable and theoretically consistent. Traditional cultural symbols in Chinese animation have a positive effect on attitudes (*β* = 0.441, *p <* 0.001) which supports H1. It has a very large and positive impact on subjective norms (*β* = 0.435, *p <* 0.001) which supports H2. It has a large positive effect on Perceived Behavioral Control (*β* = 0.364, *p <* 0.001), H3 is supported. Attitude (*β* = 0.462, *p <* 0.001), Subjective Norm (*β* = 0.199, *p <* 0.001) and Perceived Behavioral Control (*β* = 0.149, *p <* 0.05) all have significant positive impact on viewing intention, supporting H4, H5 and H6. To examine the moderating effects, a product-indicator approach was employed to create latent interaction terms, with indicators mean-centered to minimize multicollinearity. Post-hoc power analysis indicated sufficient statistical power (above 0.80) to detect these moderation effects. Cultural identity has a positive moderating impact on the connection between attitude and viewing intention (interaction term *β* = 0.148, *p <* 0.01) and effect size 0.037, supporting H4a. There is no significant effect on subjective norms and perceived behavioral control (*p* > 0.05), and therefore H5a and H6a were not supported. The simple slopes were such that on a high cultural identity (+ 1SD) the effect of the attitude to the intention to watch a film is higher. The structural model fits very well with a CMIN/df = 1.528, RMSEA = 0.035, CFI = 0.975, TLI = 0.972, R^2^ for the viewing intention = 0.512, so the model is stable.

### 5.4 Multi-group analysis and mediation effects

To examine the cross-group stability of the proposed model, a Multi-Group Analysis (MGA) was performed, with gender serving as the basis for comparison. This step is crucial to eliminate possible heterogeneity bias and guarantee the model’s applicability among various demographic groups. As illustrated in Figure 1 and Table 3, a comparison of the path coefficients was conducted between the male and female subgroups. The results of the Z-test revealed no statistically significant differences in any of the seven structural paths ( p>0.05 ). Specifically, the differences in how Traditional Cultural Symbols influence the three mediators were not statistically significant: Attitude ( Δβ=−0.052,p=0.557 ), Subjective Norms ( Δβ=−0.082,p=0.362 ), and Perceived Behavioral Control ( Δβ=−0.012,p=0.059 ). Although the *p*-value for the difference in the path from Subjective Norms to Intention was marginally above the significance threshold ( p=0.059 ), it did not reach statistical significance. These results demonstrate that the fundamental mechanism driving viewing intention remains consistent and reliable across different genders.

#### 5.4.1 Mediation analysis

**Figure 2:**
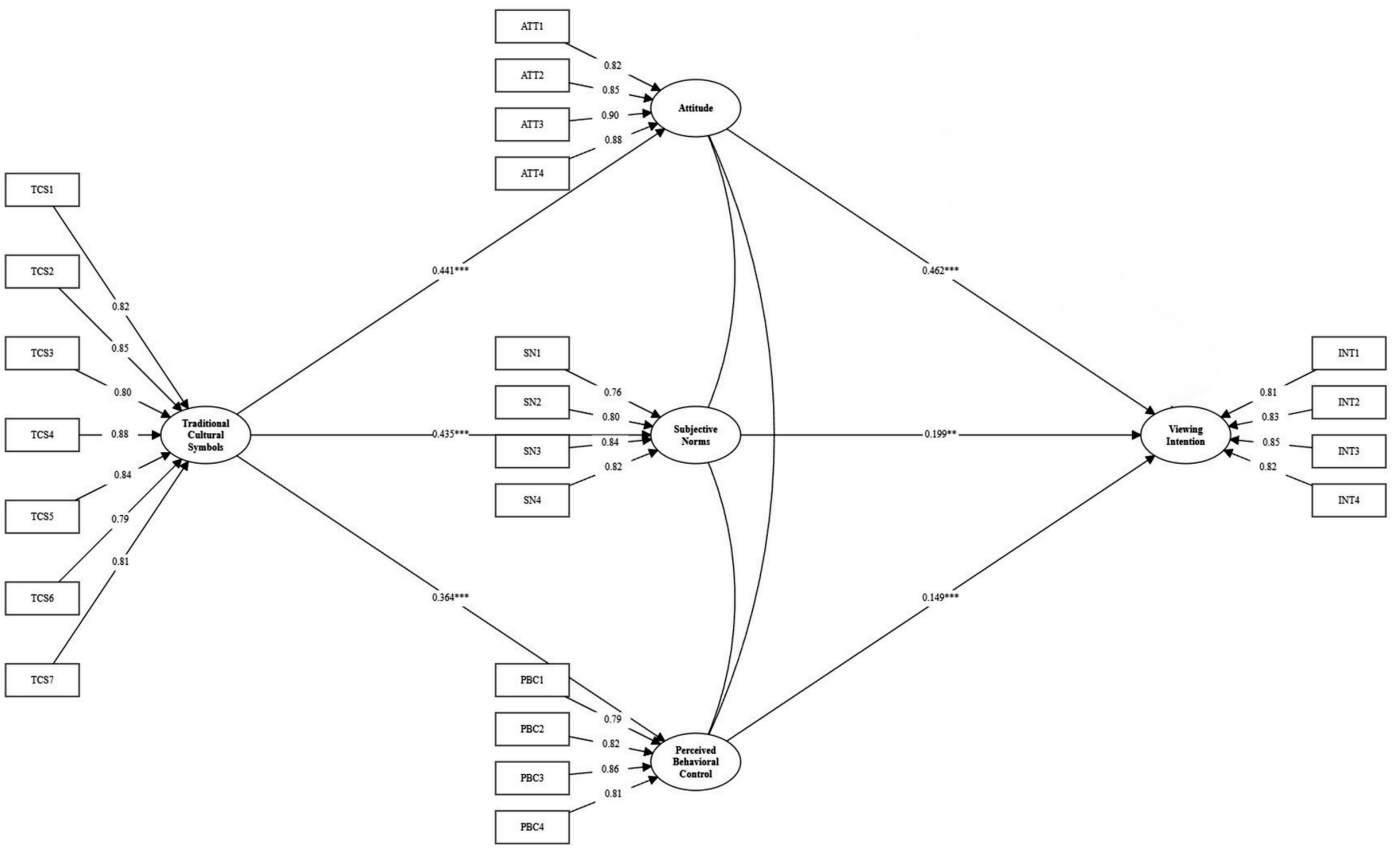
Path coefficient of the structural model.

**Figure 3:**
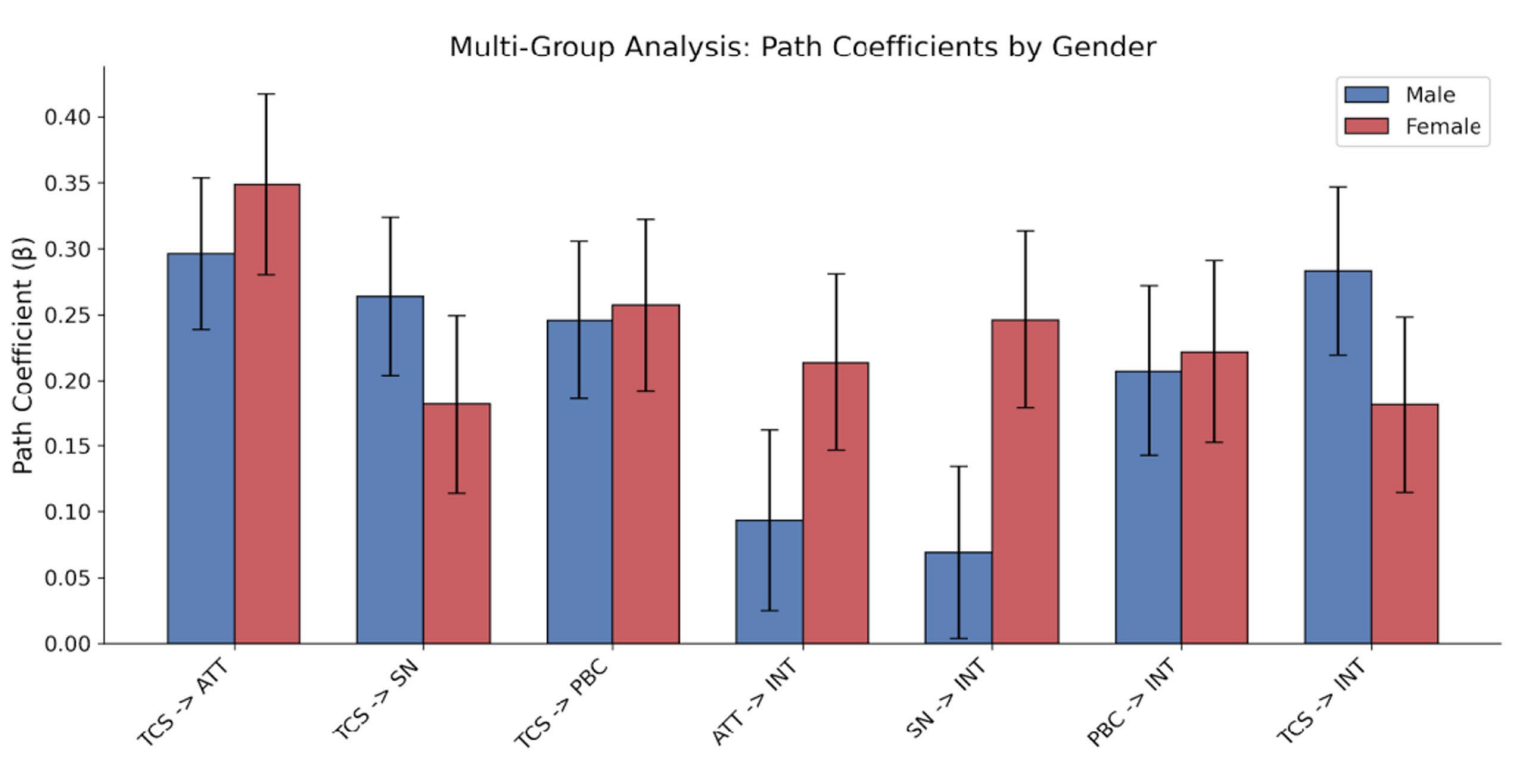
Multi-group analysis: comparison of path coefficients between male and female groups.

**Figure 4:**
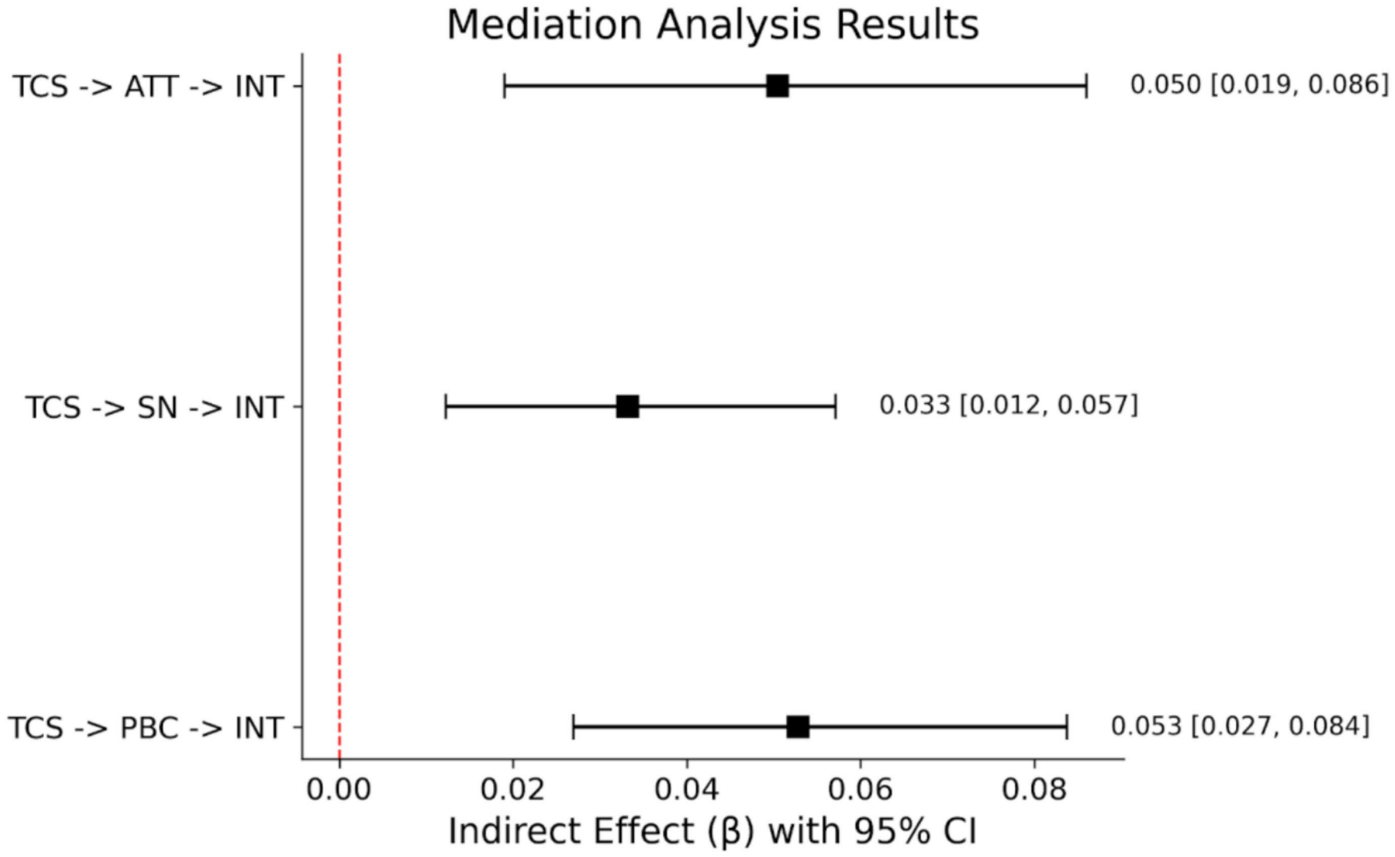
Forest plot of the specific indirect effects (with 95% CI) in the mediation model.

In order to explore the transmission path of Traditional Cultural Symbols on Viewing Intention, we use 5,000 resamples for the bootstrapping procedure to test for the mediating role of Attitude, Subjective Norms and Perceived Behavioral Control. The results are summarized in Figure 2 and Table 4. The analysis confirmed that all three indirect paths were statistically significant since their bias corrected 95% CI did not include 0: (1) Attitude: TCS → ATT → INT: *β* = 0.050, SE = 0.017, 95%CI [0.019, 0.086]. (2) Subjective norms: TCS → SN → INT: *β* = 0.034, SE = 0.012, 95%CI [0.012, 0.059]. (3) Perceived behavioral control: TCS → PBC → INT: *β* = 0.053, SE = 0.015, 95%CI [0.027, 0.084]. It can be seen that all the effects of cultural symbols are mediated by the TBP cognitive and normative components, mainly involving PBC and attitude (Figures 3, 4 and Table 5).

**Table 4:** The main fit indices of the model.

Fit indices	CMIN/df	RMSEA	TLI	CFI	GFI	NFI	AGFI
Measurement values	1.461	0.032	0.977	0.979	0.908	0.936	0.894
Standard values	<5	<0.080	>0.900	>0.900	>0.900	>0.800	>0.800

**Table 5:** The results hypotheses test.

Hypotheses	Hypothesized path	B	*β*	S. E.	C. R.	*t*	*P*	Result
H1	TCS → ATT	0.451	0.441	0.051	8.807	8.84	***	Supported
H2	TCS → SN	0.41	0.435	0.049	8.292	8.37	***	Supported
H3	TCS → PBC	0.351	0.364	0.051	6.911	6.88	***	Supported
H4	ATT → INT	0.42	0.462	0.048	8.713	8.75	***	Supported
H5	SN → INT	0.206	0.199	0.052	3.941	3.96	***	Supported
H6	PBC → INT	0.151	0.149	0.05	2.983	3.02	**	Supported

***p < 0.001, **p < 0.01, *p < 0.05.

## 6 Discussion

This study examines the influence of stimuli (i.e., traditional cultural symbols in Chinese animation movies) on organism (i.e., attitudes, subjective norm, and perceived behavioral control) and response (i.e., consumers’ willingness to watch Chinese animation films). The SOR and TPB hypotheses are tested in this empirical study. This study also examined the moderating effect of cultural identity on the link between the organism component and viewing intention. Based on the study’s results, we have drawn specific conclusions. This study also examined the moderating effect of cultural identity on the link between the organism component and viewing intention. Based on the study’s results, we have drawn specific conclusions. Beyond the specific context of Chinese animation, these findings engage with broader cultural psychology discourse by demonstrating how culturally embedded stimuli activate collective memory and social identity to drive consumer behavior in collectivistic societies, contrasting with individualistic consumption motivations.

### 6.1 Traditional cultural symbols can affect the organism

The SOR model suggests that high-level external stimuli could facilitate people’s cognitive and behavioral reactions, which is supported by this study. Especially in the context of Chinese animation, the study tends to confirm that traditional cultural symbols, as external stimuli, may influence organismic feedback, in contrast to previous research.

This study reveals that the positive cues of traditional cultural symbols have a multilevel impact on offline viewers’ intention toward Chinese animation. The traditional cultural symbols of Chinese animation, such as classical patterns and ink-wash aesthetics, carry a profound cultural connotation; thereby, they evoke significant investment in culture and emotion in the audience before viewing. The symbols have led to an increased interest in watching films in movie theaters. The viewers’ emotional ties, stimulated by cultural factors, develop into a rewarding interest that motivates the viewer to seek out more Chinese animations featuring these elements. Furthermore, animations featuring traditional cultural symbols are also unique in terms of cultural atmosphere and artistic imagery, influencing audience cognition (attitudes, perception of social norms) and beliefs about the possibility of controlling behavior. In attitudes, positive beliefs about traditional culture symbols are significantly positively related to the favorability of Chinese animation ratings.

Regarding social norms, when peer groups engage in lively discussions and appreciate traditional symbols in animations, the audience is also encouraged to learn about them. In terms of perceived behavioral control, the immersive environment of the cinema will enhance the cultural symbols conveyed through animation. The tangible presence of traditional cultural elements within the animation leads audiences to perceive that offline viewing offers a superior cultural experience, thereby enhancing their sense of control. Moderate exposure to traditional cultural symbols not only enhances consumers’ proactive engagement comprehensively but also cultivates sustained enthusiasm and immersion in cinema-going, which ensures audiences maintain enduring interest and participation in Chinese animation.

### 6.2 Attitude, social norms, perceived behavioral control can affect the response

Studies have shown that attitude, social norms and perceived behavioral control in TPB are major determinant factors in the collective theory. These variables have a positive effect on the individual’s intention to watch Chinese animation. The first is that attitude has a large bearing on behavior, which aligns with the findings of Alam et al. (2024), Ramírez-Castillo et al. (2021), and Imani et al. (2021). As an internal driving factor, the positive attitude of Chinese animation can trigger audiences’ search for information and cultivate a sense of curiosity about its content. Attitude endogenously promotes actions and influences decisions aimed at pursuing specific targets, with a significant impact on emotions and society. For example, audiences with favorable predispositions towards Chinese animation are more likely to search for related information and engage in debates about the content of these animations. In such an environment, attitudes play a significant role in determining one’s decision regarding behavior, such as going to watch movies. In addition, studies show that social norms have a positive influence on physiological reactions, which is consistent with the results of Zhang et al. (2019). Group pressure and social environment rely on societal expectations that can, in turn, influence viewing intentions. For instance, among animation fans who have norms that support the appreciation of excellent Chinese animations and the acknowledgement of their cultural value, watching Chinese animation is deemed consistent with group expectations by new members. This perception fosters a higher level of willingness to discuss and share the content, ultimately resulting in increased viewing intentions. Furthermore, the enthusiastic discussions and recommendations on Chinese animation in anime fan communities lead to conformity and compliance among members, thereby increasing their likelihood of watching related animations due to social influence.

While attitude emerged as the strongest predictor of viewing intention, perceived behavioral control significantly contributes to organismic behavior (response) as found in Jaiswal and Kant (2018) and Yue et al. (2020). This concept measures the belief that a person can be capable of performing a certain behavior. When an audience perceives themselves as being prepared to watch offline, in other words, their PBC for watching it offline becomes stronger as they believe that all necessary facilities are provided – such as transportation services to cinemas, accessibility to the scheduled screening time, and an appropriate level of readiness for planning when they visit. Thus, they have more confidence in offline viewing and stronger intentions to watch films. For instance, if movie theaters are near their homes, audiences can use an online platform to find the film’s screening schedule and then match it with their own schedule to see when they are free. Thus, they are more likely to develop a favourable intended behavior (i.e., they will intend to see animation films at the cinema). Drawing from media-environment literature, it is crucial to recognize that the strong effect of perceived behavioral control largely reflects practical accessibility factors rather than pure symbolic attachment. The consumption of animation is heavily gated by infrastructural variables—such as digital ticketing convenience, proximity to cinemas, and disposable time. Therefore, while traditional cultural stimuli trigger emotional engagement, the final behavioral execution remains fundamentally bounded by these pragmatic and structural constraints of the media consumption environment.

### 6.3 The role of cultural identity

**Figure 5:**
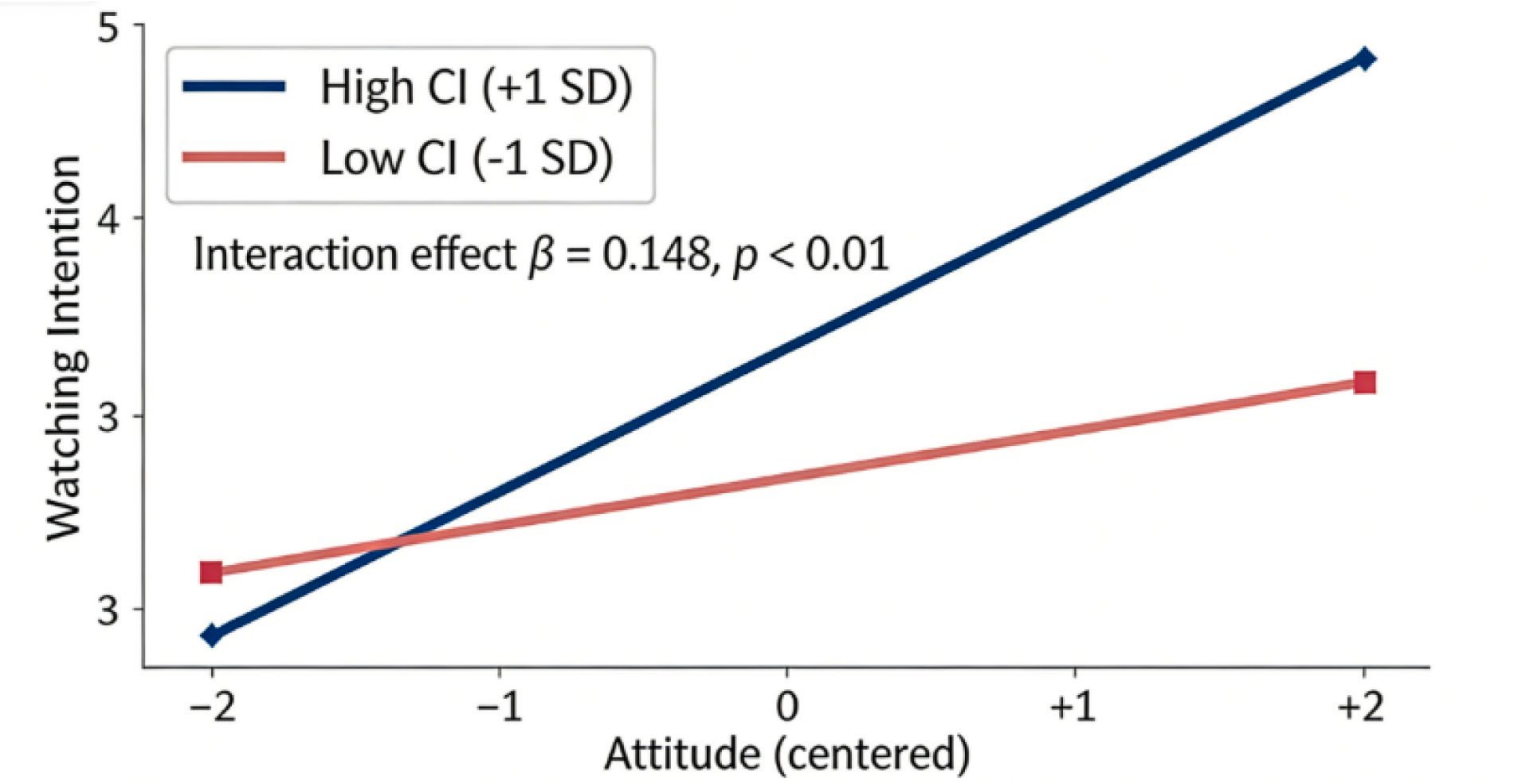
Simple slope plot of the moderating variable cultural identity (H4a).

Cultural identity refers to the degree to which an individual identifies with their culture, encompassing cultural values, beliefs, and practices. Further research results indicated that Cultural identity is a moderating variable (Figure 5), which can positively regulate the correlation of attitude and purchase intention, consistent with the findings of Liu and Zhao (2024). The effect of attitudes on purchase intention is particularly enhanced under high cultural identity (+1SD). Purchase intention increases dramatically as attitudes improve. This finding suggests that a strong cultural identity enables individuals to feel deeply connected to their culture, which in turn strengthens the relationship between attitude and purchase intention.

In contrast, consumption intention varies less at a low level of cultural identity (−1SD) with a relatively weaker impact of attitude on the variable. With the decrease of cultural identity, enthusiasm for culturally correlated consumption or behavior weakens among people, leading to the diminishing positive relationship between attitude and consumption intention. In other words, with respect to the inclination to watch Chinese animation, high and low cultural identity is also likely to moderate attitudes on viewing intention differently. They will be the audience who identify themselves through a cultural transmission, and their eagerness to know about Chinese animation will be prompted. This finding will further facilitate the participation of the masses in cultural transmission-related activities and provide solid social support for the inheritance and protection of cultural heritage. Importantly, this study found no significant moderating effect of cultural identity on the subjective norms and perceived behavioral control pathways, which is theoretically sound within a collectivistic context. Social norms often operate as pervasive environmental pressures (e.g., expectations from family or peer groups) that exert influence regardless of an individual’s personal level of cultural identity. Similarly, perceived behavioral control is heavily determined by objective infrastructural constraints (e.g., available leisure time, ticket affordability, and cinema proximity). These practical factors are relatively rigid and remain largely unaffected by the depth of a viewer’s internal cultural identification. This study contributes to a more nuanced analysis of the role of cultural identity in shaping individual consumption intentions and relevant factors by adopting a cultural identity perspective. The results of this study provide animators with useful information, helping them to better integrate cultural identity into animated content and advertising strategy, which may be related to the link between organism and consumptive intention. Furthermore, these findings resonate with broader cultural psychology literature beyond the Chinese context. In collectivistic societies, cultural symbols serve as potent priming mechanisms for collective memory and social cohesion. Unlike individualistic Western media contexts, where viewing intentions are frequently driven by personal gratification and distinctiveness, the consumption of traditional cultural products in this context is heavily mediated by the reinforcement of shared cultural heritage and group identification.

### 6.4 Robustness check and gender universality

While cultural identity accounts for the diversity in viewing intensity among individuals (as noted earlier), it is equally crucial to verify the structural consistency of the model across various demographic groups. To confirm this, the results of the Multi-Group Analysis (MGA) revealed no statistically significant differences in the path coefficients between the male and female groups ( p>0.05 ). This demonstrates that the psychological pathway ( TCS→TPB→Intention ) remains consistent and unchanged across different genders.

This discovery enhances our moderation outcomes by shedding light on a “dual mechanism”: Psychographic factors, such as cultural identity, influence the strength of the relationships, while demographic factors, like gender, do not change the structural arrangement of the mechanism. These findings indicate that the appeal of Chinese-style animation is rooted in a shared cultural memory, which resonates uniformly across different genders. Hence, the market strategy ought to be twofold: on one hand, leveraging cultural symbols to spark widespread resonance (gender universality); on the other hand, targeting specific segments according to their level of cultural identification (psychographic moderation).

## 7 Conclusions and implications

### 7.1 Research conclusions

This study integrates the Stimulus-Organism-Response (SOR) and Theory of Planned Behavior (TPB) to construct and validate a serial mediation model, systematically revealing the formation mechanism of Chinese animated film viewing intention. Empirical analysis shows that traditional cultural symbols, as key external stimuli, significantly and positively influence audiences’ attitudes, subjective norms, and perceived behavioral control—three psychological variables that further drive viewing intention. Cultural identity exerts an asymmetric moderating effect, significantly positively moderating only the attitude → viewing intention path but not the paths between subjective norms, perceived behavioral control and viewing intention. This study makes three main theoretical contributions: first, it is the first to explicitly conceptualize “traditional cultural symbols” as core external stimuli (S) for cultural virtual products under the SOR framework, breaking the theory’s application limitations in physical product consumption research and expanding its scope. Second, it constructs and validates an integrated SOR-TPB serial mediation model, clarifying the complete path of “cultural symbol input → psychological mechanism transmission → viewing intention output” and filling the research gap regarding the transformation mechanism of external cultural cues into consumption intention. Third, it reveals the asymmetric moderating effect of cultural identity on the viewing intention formation paths. Rather than challenging TPB outright, this finding refines the model’s contextual application. It suggests that cultural identity primarily amplifies affective and personal evaluations (attitude), while having a limited impact on socially driven factors (subjective norms) or practically constrained resources (perceived behavioral control). This indicates that in collectivistic settings, social pressures and objective constraints remain robust regardless of individual cultural identification.

### 7.2 Theoretical implications

Firstly, by integrating the SOR and TPB theories, this research presents a theoretical model that illustrates the mechanism by which traditional cultural symbols influence participation in Chinese animation. It also contributes to the theoretical discourse of animation design and promotes people’s critical engagement with traditional cultural symbols when designing cartoon characters. Hitherto, TPB research has mainly focused on investigating purchase intention for tangible products. In contrast, this study, which is conducted in the field of viewing Chinese animation films, uses SOR theory to explore consumers’ watching behavioral motivation from the perspective of cultural identity. This procedure offers an innovative theoretical perspective on the consumption of cultural goods. It also fills in the gaps of previous empirical analyses, thereby contributing to a more comprehensive theoretical understanding of the effects of traditional cultural symbols design.

Secondly, it is also beneficial for empirical methods to apply systematic validation of the existing theoretical research and to overcome its limitations. Results revealed that cultural identity has a significant positive moderator role between attitude and viewing intention. This finding suggests that audiences with a high level of cultural identity are more likely to change their positive attitudinal dispositions towards traditional cultural symbols when they actually encounter them. This research empirically supports the theory of the conversion mechanism between emotion and cognition in cultural consumption, thereby promoting our understanding of the logic underlying individual decision-making in cultural consumption.

Thirdly, the empirical evidence supports differentiated influence paths, as all three TPB elements significantly influence viewing intention, albeit to varying degrees. The impact of PBC, attitude and SN on viewing intention declines in this order. This phenomenon suggests that ranking separate psychological mechanisms should be a priority in studying the consumption of animation films.

### 7.3 Managerial implications

Firstly, strengthening public education on traditional culture and enhancing the quality of film are our key tasks. To sum up, organismic factors (i.e., attitudes, subjective norms, perceived behavioral control) are one of the significant factors that affect consumers’ intentions to view films. Perceived behavioral control has a significant and manifest effect on the intention to go to the cinema, so improving the convenience of cinema antecedents, such as manageability at home, is important. During the offline cinema watching process, it is suggested to integrate online and offline ticketing channels. This suggestion incorporates furthering relationships with some of the biggest ticketing platforms, adding dedicated booking lanes for members and creating flexible price bands (including student concessions and deals). Cinema operators ought to focus on cultivating an immersive cultural atmosphere that strengthens the audience’s sense of ritual and engagement, instead of merely emphasizing physical facilities, while also offering quick ticket verification and self-service concessions.

Secondly, it is essential to comprehend the role that cultural identity plays in moderating, and understanding a product’s cultural heritage attributes is crucial. Although the relationship between such modifications and the moderation effect of cultural identity on consumer subjective norms and perceived behavioral control is not empirically supported, it has a positive influence on attitudes. More specifically, the higher one’s cultural identity, the more likely one is to hold positive beliefs about animations featuring traditional cultural symbols. Furthermore, production companies need to adhere to the basic concept of “strengthening attitudes through cultural symbols” in the design of animation. For instance, they should adopt traditional cultural symbols like high familiarity plus high recognition. It would be suggested that user polls be taken to test consumer recognition and preferences of any symbol, for example, a conventional motif or classic story intellectual property. Then the popular symbols are inserted into character creation, scene arrangement and narrative theme to further contribute to the viewers’ positive evaluation of that work. In addition, developing products closely related to the cultural symbols of the animation and organizing offline immersive experiences (for example, making traditional crafts with an animated background) can be beneficial for enhancing consumers’ positive feelings toward the animation. Lastly, it is essential to identify key core elements in traditional culture that resonate strongly with today’s values and conscientiously incorporate these into our animation storyline. This methodology directs cultural identification to shift attitudes through “value resonance,” thus preventing the use of symbols where their meaning is superficial, and ensuring that a positive correlation exists between the popularity of traditional culture and the audience’s endearment.

Furthermore, there should be room for upgrading online platforms to control picture quality and bullet-screen interactive permissions; individually customized options (e.g., speed change and mirroring) need to consider the needs of viewers with different habits. After watching, cinemas should solicit feedback through survey questionnaires and face-to-face sessions, while online platforms must allow comments to flourish. Communities specific to each movie need to be created, with staff responding immediately to input from the audience, so they feel as if their input is taken seriously. Additionally, attitude is a crucial factor in predicting viewing intention. Producers are encouraged to thoroughly explore traditional culture and incorporate it into animation plots, character design, and scene establishment. Through “cultural resonance,” they can amplify the goodwill that audiences hold toward the work. Additionally, aesthetically effective animation contributes to the value of culture through aspects such as visual texture, narrative consistency, voice acting, and music arrangement. This method enables viewers to appreciate the cultural value and the works themselves, thereby promoting a more intentional viewing experience.

Subjective norms have comparably weaker impact on viewing intention, suggesting that marketing strategies should be adjusted to avoid over-reliance on social endorsement. Rather, focus should be on the indirect effect of impact through cultural identity. In particular, social media resources can be employed to start a conversation about “the cultural meaning of traditional culture animation”, prompting audience members to share their own understandings and associations with the cultural symbols illustrated in the artwork. It is with such a “promote cultural topics”, rather than on “opinion leader support,” that they indirectly influence the attitude within their social milieu toward the work. To achieve this, it could set up a variety of offline cultural salons that focus on traditional culture animation and work with the audience to provide in-depth interpretations of profound culture, fostering creative interaction. Attempting to create cultural resonance and word-of-mouth propagation indirectly through this way effectively taps into the implicit weight of subjective norms.

### 7.4 Research limitations and future research

It provides a detailed view of consumer attitudes toward Chinese cartoons; however, it also has several limitations that need to be addressed in future research. First, the use of quantitative research methods hindered our capability to further depict in depth the great diversity and complexity of participants. In disproof of the argument to support the above-mentioned threats in future studies, there is an opportunity to gather qualitative information, such as interviews, along with a mixed-method approach and cross-correlate findings, results and conclusions of this research. Second, regarding sampling constraints, the data was restricted to participants in China and is heavily concentrated among young adults aged 18–25 (74.15%). While this demographic represents the core consumer base for Chinese animated films, this age concentration limits the generalizability of the findings across older age groups, who may possess different cultural identity dynamics and media consumption habits. This limitation should be mitigated by future studies that employ stratified sampling to investigate age-related differences and collect samples internationally. Furthermore, using stratified or multistage sampling techniques would enhance the generalizability and relevance of these results.

Furthermore, using stratified or multistage sampling techniques would enhance the generalizability and relevance of these results. The categorization of Chinese animated traditional culture symbols was not refined enough in this study. It remains to be seen how intrinsic categories of traditional cultural symbols in Chinese animation films can be further investigated in future studies. Adding other traditional cultural symbols into measurement, such as ethnic music and traditional costumes, could contribute to the improvement of validity and generalizability in this study. Besides, this study only focuses on the intention of consumer to watch movies but not on their actual behavioral strategies. In the future, research needs to discuss the gap between the interest in viewing Chinese animation films and the actual viewing behavior. Fourth, regarding methodological constraints, this study employed a cross-sectional design, which limits the ability to establish strict causal relationships between traditional cultural stimuli, psychological mechanisms, and viewing intentions. Future research should utilize longitudinal designs or experimental approaches to better validate the causal inferences proposed in this model.

## Data Availability

The original contributions presented in the study are included in the article/supplementary material, further inquiries can be directed to the corresponding authors.
